# Development of Foot-and-Mouth Disease Virus-Neutralizing Monoclonal Antibodies Derived From Plasmablasts of Infected Cattle and Their Germline Gene Usage

**DOI:** 10.3389/fimmu.2019.02870

**Published:** 2019-12-06

**Authors:** Kun Li, Sheng Wang, Yimei Cao, Huifang Bao, Pinghua Li, Pu Sun, Xingwen Bai, Yuanfang Fu, Xueqing Ma, Jing Zhang, Dong Li, Yingli Chen, Xuerong Liu, Fanglan An, Faju Wu, Zengjun Lu, Zaixin Liu

**Affiliations:** ^1^State Key Laboratory of Veterinary Etiological Biology, Lanzhou Veterinary Research Institute, Chinese Academy of Agricultural Sciences, Lanzhou, China; ^2^China Agricultural Vet Biology and Technology Co. Ltd., Lanzhou, China

**Keywords:** cattle, foot-and-mouth disease virus, broadly neutralizing antibodies, antigenic character, single B cell antibody

## Abstract

Cattle are susceptible to foot-and-mouth disease virus (FMDV), and neutralizing antibodies are critical for protection against FMDV infection in this species. However, more information is needed on the host specific antigenic structure recognized by the FMDV-specific monoclonal antibodies (mAbs) and on the functional properties of the mAb that are produced in the natural host, cattle. Herein, we characterized 55 plasmablast-derived mAbs from three FMDV-infected cattle and obtained 28 FMDV-neutralizing antibodies by the single B cell antibody technique. The neutralizing mAbs (27/28) mainly recognized conformational epitopes that differ from the well-characterized immunodominant antigenic site 1 of FMDV as defined by murine mAbs. Of these FMDV-neutralizing mAbs, 13 mAbs showed intra-type broadly neutralizing activity against the three topotypes of FMDV serotype O (ME-SA, SEA, and Cathay topotypes). Moreover, all these intra-type broadly neutralizing antibodies competed with sera from FMDV infected or vaccinated cattle, which indicates their binding to native dominant epitopes, as revealed by a blocking ELISA. We further analyzed the germline V(D)J gene usage of the 55 FMDV-specific mAbs and found cattle IgG antibodies containing ultralong HCDR3 were exclusively restricted to usage of the germline gene segment V_H_ 1-7^*^02. In addition, the restricted germline gene segments of V_H_ 1-7^*^02 and V_L_1-47^*^01 or 1-52^*^01 pairing were observed in all IgG antibodies with ultralong HCDR3. Furthermore, antibodies with longer HCDR3 were more inclined to display FMDV-neutralizing activity. This study presents a novel method for screening FMDV-specific cattle mAbs which then provide the most useful tools for studying FMDV antigenic structure and variation.

## Introduction

Foot-and-mouth disease (FMD) is a highly contagious and economically important viral disease of cloven-hoofed livestock, including cattle, sheep and pigs. The causative agent is foot-and-mouth disease virus (FMDV), an *Aphthovirus* in the Picornaviridae, and appears as seven serotypes (i.e., O, A, C, Asia1, SAT1, SAT2, and SAT3) and several topotypes, with uneven geographic distributions. FMDV serotype O has been a major threat to animal husbandry in recent years in China. Four lineages in the three topotypes of FMDV type O, namely, Cathay, Middle East-South Asia (ME-SA), and South-East Asia (SEA) topotypes, are introduced and currently circulating in China, which makes the situation rather complicated. Antigenic variation among these topotypes has been investigated in recent years (1, 2). However, detailed differences in antigenic structure of these topotypes are still not delineated. Monoclonal antibodies (mAbs) recognizing neutralizing epitopes could provide important keys to the basis of this antigenic variation.

There is good evidence that humoral responses play a major role in protection against FMDV infection in natural hosts (3, 4). As a natural host of FMDV, cattle have a distinct composition of immunoglobulin (Ig) repertoire compared to other vertebrates which display restricted lengths of the third heavy chain complement determining regions (HCDR3s) with an average of 12–16 amino acids (aa) in length (5). However, cattle produce antibodies containing HCDR3s with an average length of 26 aa, including an ultralong subset that can exceed 60 aa (6, 7). The proportions of kappa (κ) and lambda (λ) light chains in cattle Ig are 5 and 95%, respectively, whereas those of rodents are 95 and 5%, respectively (8). These unique characters of Ig sequences make cattle a promising host for producing high avidity and broadly neutralizing antibodies (bnAbs), exemplified by the rapid elicitation of bnAbs to HIV by immunization of cattle; these bnAbs contained ultralong HCDR3s that were responsible for their serological breadth and potency (9). However, it is currently unknown whether the ultralong HCDR3s are responsible for their high avidity and broadly virus neutralization against FMDV.

Up to now, monoclonal antibodies (mAbs) selected from mouse hybridomas have been extensively used to investigate the antigenic profile of FMDV. As revealed by these mouse mAbs, five functionally-independent neutralizing antigen sites (3–7) have been identified on the capsid surface of FMDV serotype O. Site 1 is linear and trypsin sensitive, which encompasses the G-H loop and the C terminus of VP1, with critical residues at positions 144, 148 and 150, and 208 that affect antibody binding. However, other identified sites (i.e., sites 2–5) are all conformational and trypsin resistant. Site 2 is defined by mutations in the VP2 B-C or E-F loops, involving critical aa residues at positions 70–73, 75, 77, and 131. Critical residues at positions 43 and 44 in the VP1 B-C loop, and at position 58 in the VP3 B-B “knob” contribute to site 3 and site 4, respectively. Site 5 contains at least a functionally independent neutralizing epitope that involves a specific mutation at position 149 in the G-H loop of VP1, which is distinct from site 1 despite part of the G-H loop is encompassed (10–14). More recently, a new neutralizing epitope that involves the position 192 of VP2 at the 3-fold axis was reported (15). FMDV serotype O specific cattle mAbs selected from a mouse × cattle hetero-hybridoma were used to compare antigenic features defined by mouse mAbs, and these cattle mAbs recognize identical antigen sites 2, 3, and 4 but not antigen sites 1 and 5 (16). However, due to the inherent character of cattle-derived mAbs, the virus-neutralizing features of ultralong HCDR3-associated mAb need to be further surveyed using large numbers of mAbs derived from the natural host of cattle.

MAbs are produced from a single B-lymphocyte clone and can bind to a specific epitope. The first mAb was developed in the mouse by a hybridoma technique in 1975 (17). With the improvements in genetic sequencing and biomedical instrumentation, new methods for generating mAbs have been reported in recent years, including B cell immortalization (18, 19), phage display (19), and single B cell antibody technique (20). All these methods have different merits and were chose to develop mAbs based on different purposes and conditions.

**Figure 1:**
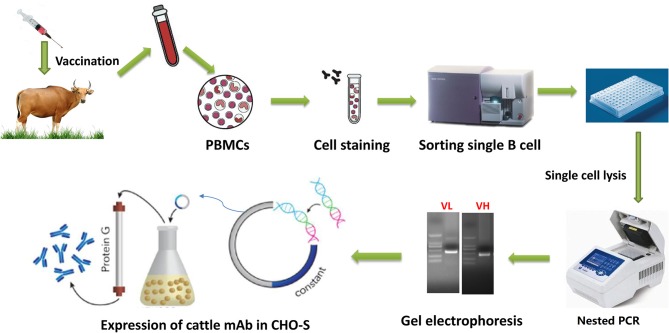
Workflow of cattle mAb production using singe B cell antibody techniques. Cattle were sequentially immunized with different FMDV serotype O strains, and then peripheral blood was collected to isolate PBMCs. The isolated PBMCs were stained with a panel of mouse anti-cattle CD21, anti-cattle IgM, and biotinylated antigen, followed by incubation with APC labeled anti-biotin secondary antibody. The single antigen-specific plasmablast was sorted into 96 well-plate with lysis buffer and immediately reverse-transcribed into cDNA. The paired VH and VL sequences was respectively amplified by nested PCR and then sequenced. The variable regions were codon optimized and cloned into pcDNA3.4 expression cassette containing cattle IgG constant region coding sequences. The recombinant mAbs were expressed in CHO-S and purified by affinity and further molecular sieve chromatography.

This study aimed to produce diverse mAbs in natural host by single B cell antibody technique, in order to explore the antigenic variation of FMDV and to shed light on FMDV host-specific antigen site. The results show that diverse antibodies can be obtained by this single B cell antibody technique (procedures are showed in Figure 1), so providing abundant tools for exploration the antigenic variation of FMDV type O. Furthermore, rearrangements in antibody genes of FMDV-specific mAbs were analyzed, and restricted germline gene segment usage was observed in the ultralong HCDR3 containing antibodies. These data enrich our knowledge on the diversity of antibodies and their mechanism of production.

## Results

### Immune Response of Cattle Sequentially Infected With FMDV Serotype O

**Figure 2:**
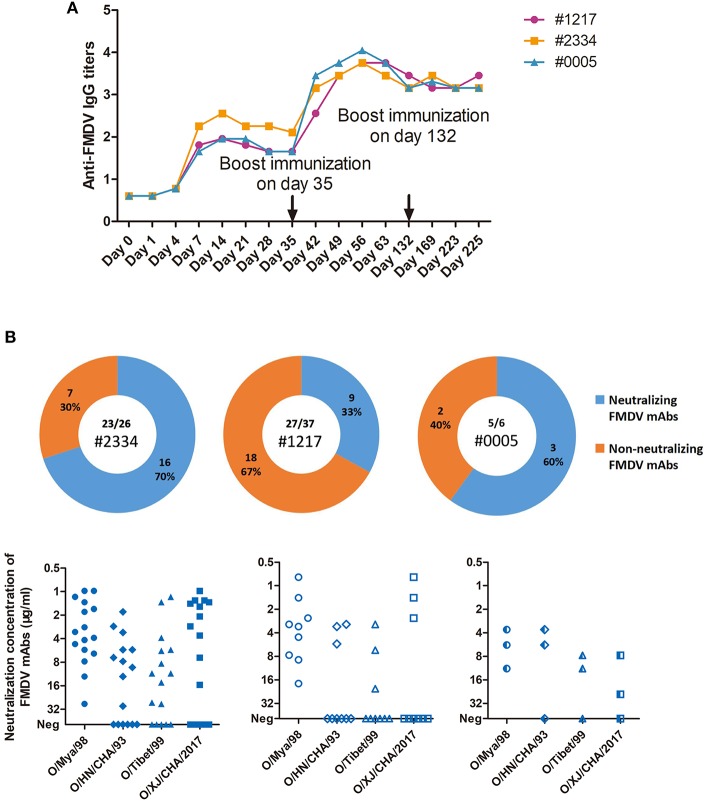
Cattle IgG antibody titers against FMDV in sera, and the virus-specific plasmablast-derived mAbs from 3 infected cattle. **(A)** A total of 3 cattle (#1217, #1234, and #0005) were sequentially infected with O/Mya/98, O/HN/CHA/93, and O/Tibet/99 strains at days 0, 35, and 132. Sera were pooled at days 0, 7, 14, 21, 28, 35, 42, 49, 56, 63, 132, 169, 223, and 225 after first infection. IgG titres were expressed as the reciprocal log10 of serum dilutions that yielded 50% of the absorbance value of the negative control wells in LPB-ELISA. IgG titres less than the sensitivity of the assay (0.9) were adjusted to 0.6 in the figure. **(B)** IgG plasmablasts from the 3 cattle were collected to produce mAbs, which were specific for FMDV in 23 out of 26 cells from #2334, 27 out of 37 cells from #1217, and 5 out of 6 cells from #0005.

As revealed by the results of LPB-ELISA (Figure 2A), the humoral immune responses in the three cattle were detected on the fourth day and peaked on day 14 after initial infection with the FMDV O/Mya/98 (SEA topotype) strain. After a boost immunization with FMDV O/HN/CHA/93 (Cathay topotype), IgG titers increased rapidly and reached their highest levels on day 56. However, the third immunization with FMDV O/Tibet/99 (ME-SA topotype) failed to further promote increase in IgG level, and these high titers persisted for at least 4 months. The antibody titers against the four strains of the three topotypes of FMDV serotype O were detected by virus-neutralizing test (VNT). A broad antibody spectrum against the three topotypes of FMDV serotype O were observed in the three cattle (Table S1), which was initiated from the first infection to the third immunization. To isolate the FMDV-specific plasmblasts, peripheral blood mononuclear cells (PBMCs) from cattle were generally collected after the third, depending on the antibody level. The cattle number and time points for isolation of PBMC are listed in Table S2, corresponding to each mAbs.

### Distribution and Proportion of FMDV-Specific Plasmablasts in Infected Cattle

**Figure 3:**
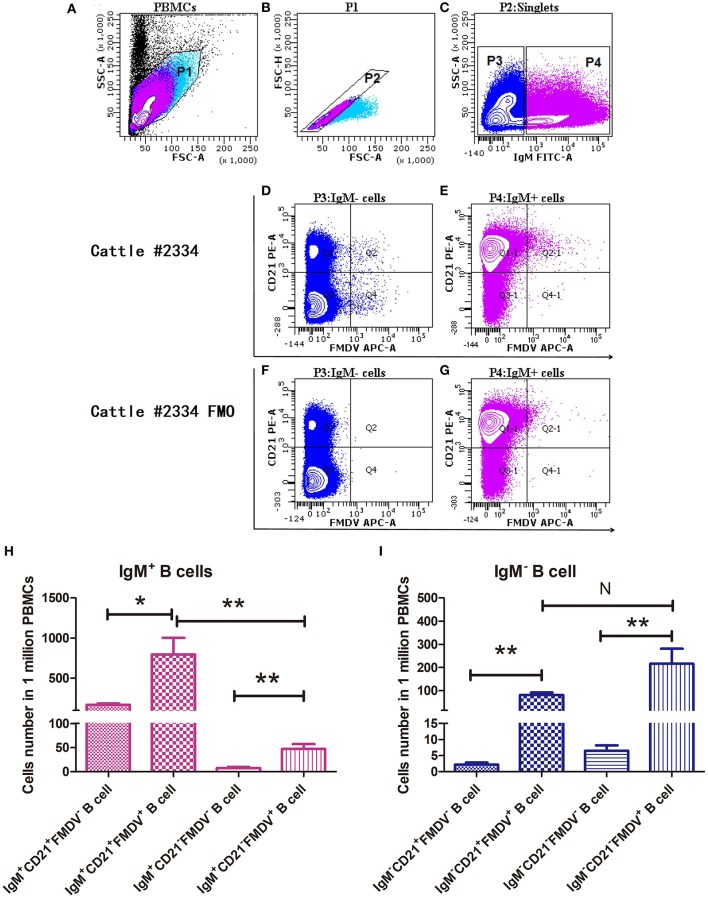
Confirmation of the phenotype and proportion of FMDV-specific plasmablasts in the peripheral blood by flow cytometry. Cattle PBMCs **(A)** were analyzed by three-color flow cytometry and gate 1 **(B)** was selected to exclude cells debris with lower values of SSC-A and FSC-A, and further analyzed to gate singlets **(C)** based on diagonal streak of the FSC-A and FSC-H plot. The IgM^−^ B cells were gated to check the distribution of CD21 and FMDV-specific cells, in the presence **(D)** or absence **(F)** of FMDV antigen. Similarly, IgM^+^ B cells were gated to check the distribution of CD21 and FMDV-specific cells, in the presence **(E)** or absence **(G)** of FMDV antigen. One million PBMCs were collected per sample. The distribution and phenotype of FMDV-specific plasmablasts in the IgM^+^ B cells **(H)** and the IgM^−^ B cell **(I)** population were analyzed. The differences between two groups were marked with asterisks in *t*-test; wherein the alpha values were labeled as NS (*P* > 0.05), ^*^P < 0.05 and ^**^P < 0.01.

According to the FACS results shown in Figure 3, FMDV-specific plasmablasts were a minor population in the peripheral blood at a frequency of <0.1% PBMCs. At least 1 × 10^6^ PBMCs were analyzed to check the distribution and proportion of FMDV-specific plasmablasts in infected cattle. The majority of FMDV-specific plasmablasts are distributed in the IgM^+^ B cell population, including the majority population of IgM^+^CD21^+^FMDV^+^ B cells (0.0796% ± 0.0207%) and the minority population of IgM^+^CD21^−^FMDV^+^ B cells (0.0047% ± 0.0009%; *n* = 6, Figure 3B). Within the IgM^−^ B cells, which might consist of either IgG^+^ or IgD^+^ B cells, the IgM^−^CD21^+^FMDV^+^ B and IgM^−^CD21^−^FMDV^+^ B cells accounted for about 0.0216 ± 0.0065% and 0.0081 ± 0.0010%, respectively (*n* = 6, Figure 3C). Owing to the phenotypic character of FMDV-specific plasmablasts, single IgM^−^CD21^+/−^FMDV^+^ B cells were sorted for the amplification of the IgG BCR variable gene and development of FMDV-neutralizing mAbs.

### Production of Plasmablast-Derived FMDV-Specific mAbs in Cattle

**Figure 4:**
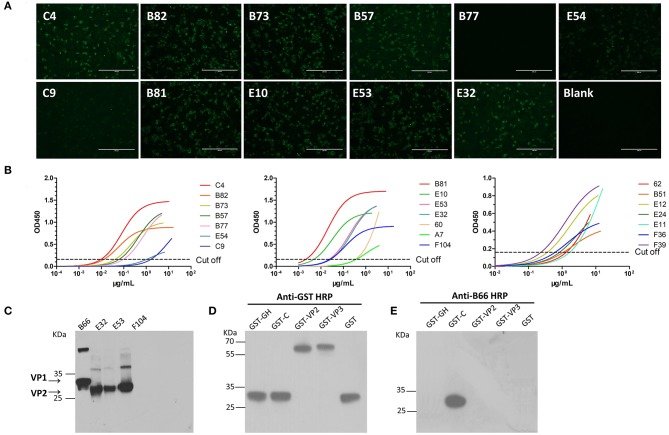
Characterization of FMDV-specific mAbs. The reactivity of cattle mAbs were checked by IFA, indirect ELISA and WB. The IFA **(A)** was performed using BHK cells infected with the O/Mya/98 strain, and the working concentration of the tested cattle mAbs was 5 μg/ml, followed by incubation with goat anti-cattle FITC (diluted 1:5,000 in PBS). The indirect ELISA **(B)** were carried out by incubation with different concentrations of 0–20 μg/ml of the tested mAbs with FMDV 146S antigen, followed by probing with anti-His HRP (diluted 1:6,000 in PBS). The lysis of 146S antigen **(C)** and GST-fused antigens **(D)** of FMDV were subjected to 12% SDS-PAGE and electrophoretically transferred to a nitrocellulose membrane. The membrane was incubated with tested mAbs and then probed with a 1:5,000 dilution of HRP-conjugated goat anti-cattle IgG (Sigma-Aldrich, USA). **(E)** B66 stained with GST-C antigen and showed the binding with VP1 C-termial of FMDV.

The pairs of γ heavy chain (Figure S1A) and λ light chain (Figure S1B) variable regions (designated as VH and VL, respectively) of cattle IgG were successfully amplified from sorted single plasmablasts by nested PCR and subsequently confirmed by DNA sequencing. The complete cattle IgG mAbs were successfully expressed in CHO-S cells and then purified, as shown by reduced (Figure S1C) and non-reduced SDS-PAGE (Figure S1D). A total of 55 FMDV-specific mAbs were produced from the three cattle. Among them, 23 out of 26 B-cell antibodies (89%) were obtained from #2334, 27 out of 37 B-cell antibodies (73%) from #1217, and 5 out of 6 B-cell antibodies (83%) from #0005 cattle (*P* = 0.8782; χ^2^ test), as shown by staining of virus-infected cells by indirect immunofluorescent assay (IFA) and binding of the purified virus antigen by ELISA (Figures 4A,B, Table S2) and VNT. Of the 55 FMDV-specific mAbs, only four recognized denatured FMDV capsid proteins by western-blot (WB; Figure 4C). This result suggests that these FMDV-specific mAbs predominantly recognize the conformational epitopes of the viral capsid.

Of the 55 FMDV-specific antibodies, 28 FMDV-neutralizing mAbs were identified (Figure 2B). No significant difference in the percentage of neutralizing mAb clones among the three cattle was observed, and 70% of FMDV-specific IgG plasmablasts were found in #2334, 33% were found in #1217 and 60% were found in #0005 (*P* = 0.3317, χ^2^ test). However, a large proportion of the neutralizing clones from #2334 (8 out of 16; 50%) and #0005 cattle (2 out of 3; 67%) showed high-potency cross-reactivity with the three topotypes of FMDV serotype O.

### Potency and Breadth of FMDV-Neutralizing mAbs

To check the neutralization breadth and potency of the natural host-derived FMDV-neutralizing mAbs, they were tested against the four representative strains of FMDV (O/HN/CHA/93; O/Tibet/99; O/Mya/98; and O/XJ/CHA/2017) from the three currently epidemic topotypes (i.e., ME-SA, SEA, and Cathay) of serotype O in China (Table 2). The 28 neutralizing mAbs obtained exhibited variable neutralizing breadth and potency against four representative FMDV strains, but all of them were able to neutralize O/Mya/98 virus. We believe that this results from using this virus as a bait antigen to bind FMDV-specific single B cells, of which, 13 mAbs (i.e., B57, B73, B77, B82, C4, C9, E46, E54, F18, F128, F145, F150, and F169) showed intra-type bnAbs activity as indicated by their ability to neutralize the four representative strains of FMDV serotype O.

**Table 1:** Nested-PCR primers used for amplification of variable regions of cattle IgG.

**Primers**	**^a^Sequences**	**^b^Ta (°)**
Ig γ chain outer-Forward:	CCCTCCTCTTTGTGCTSTCAGCCC	58/60
Ig γ chain outer-Reverse:	GTCACCATGCTGCTGAGAGA	60
Ig γ chain inner-Forward:	AGAGGRGTYBTGTCCCAGG	55
Ig γ chain inner-Reverse:	CTTTCGGGGCTGTGGTGGAGGC	55
Ig λ chain outer-Forward:	CACCATGGCCTGGTCCCCTCTG	56
Ig λ chain outer-Reverse:	AAGTCGCTGATGAGACACACC	56
Ig λ chain inner-Forward:	TGGGCCCAGGCTGTRCTG	55
Ig λ chain inner-Reverse:	GCGGGAACAGGGTGACCGAG	55

a *Degenerate bases were synthesized in these sequences, including S = C or G, Y = C or T, and R = A or G*.

b *Annealing temperature*.

**Table 2:** Evaluation of cattle-derived FMDV-neutralizing mAbs for neutralization and potency.

**Clone**	**VNT**			
	**O/HN/CHA/93 (CATHAY topotype)**	**O/Tibet/99 (Panasia subset of ME-SA topotype)**	**O/Mya/98 (SEA topotype)**	**O/XJ/CHA/2017 (IND2001 subset of ME-SA topotype)**
A19	>50	>50	17.87	>50
A35	>50	>50	1.43	1.43
B55	>50	>50	8.89	>50
B57	5.56	20.83	2.60	2.60
B66	>50	>50	3.13	>50
B73	7.00	26.25	3.28	7.00
B74	>50	>50	7.81	15.63
B77	7.81	3.91	3.91	0.98
B82	9.33	8.33	4.17	2.08
B83	>50	>50	12.22	>50
C4	12.22	5.73	2.86	1.43
C5	>50	>50	4.69	>50
C9	29.17	14.58	1.82	3.65
E18	3.13	3.13	0.78	>50
E34	12.50	>50	3.13	>50
E46	5.73	11.46	5.73	24.44
E50	>50	>50	11.46	>50
E54	3.65	7.78	3.65	7.78
F28	3.33	6.67	3.33	0.78
F41	>50	>50	7.72	>50
F53	>50	>50	4.55	>50
F103	>50	>50	6.22	>50
F128	5.56	11.11	0.98	1.30
F136	>50	>50	1.67	>50
F145	1.82	1.37	1.37	1.37
F150	3.33	27.27	27.27	1.56
F166	>50	1.17	1.17	>50
F169	2.78	5.56	5.56	2.78

*The mAbs were tested on four strains belonging to three topotypes of FMDV type O. Values are neutralization IC_50_ in μg/ml. An IC_50_ value of 50 μg/ml was used as a cut-off for neutralization and >50 μg/ml was determined as no virus neutralizing activity. The IC_50_ value between 0–1 μg/ml was marked as purple, 1–5 μg/ml as blue, and 5–10 μg/ml as red, and 10–25 μg/ml as orange, and 25–50 μg/ml as green*.

The neutralizing antibody titer was expressed as a 50% inhibitory concentration [IC_50_ (9)]. Five mAbs (B77, E54, F28, F145, and F169) had potent neutralization with IC_50_ of <10 μg/ml. Of these the mAb F145 showed the most potent neutralization with IC_50_ around 1 μg/ml against 4 strains. The mAbs E18 and F128 displayed ultra-potent neutralization with IC_50_ <1 μg/ml against O/Mya/98 strain (the SEA topotype), and the mAbs F28 and B77 displayed ultra-potent neutralization against the O/XJ/CHA/2017 strain (the IND2001 subset of ME-SA topotype). Antigenic variation among different topotypes or even different strains could be observed from the data listed in Table 2. Four mAbs neutralized only one of the two strains in the ME-SA topotype, as revealed by the mAbs A35 (IC_50_ <5 μg/ml), E18 (IC_50_ <5 μg/ml), B74 (IC_50_ <25 μg/ml), and F166 (IC_50_ <5 μg/ml), indicating some differences in the neutralizing epitopes between the India 2001 and PanAsia subsets of ME-SA topotype of FMDV type O.

### Immunodominant Antigenic Profile of FMDV Serotype O Specified in the Natural Host Was Revealed by Intra-Type bnAbs

**Figure 5:**
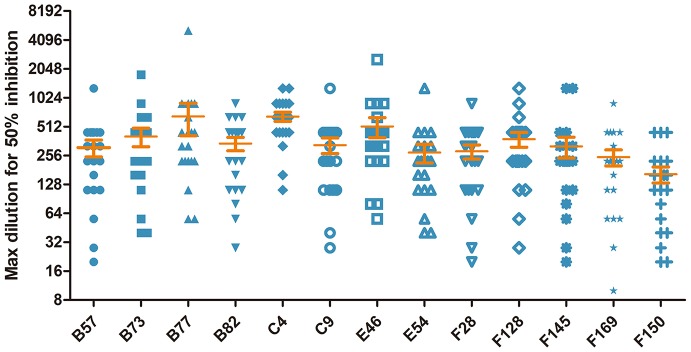
Evaluation of the cattle-derived intra-type bnAbs-based immunodominant antigen sites of FMDV serotype O using a blocking ELISA. The 13 intra-type bnAbs were respectively coated on a 96-well plate overnight, and 20 serum samples consisting of 10 from vaccinated cattle and 10 from experimentally infected cattle were incubated with variable diluted titers of FMDV 146S antigen. The result corresponding to each tested mAb was expressed as the serum dilution that yielded 50% of the absorbance OD_450_ value of the PBS control wells.

To shed light on the antigenic profile of FMDV serotype O specified in the natural host, these 28 cattle-derived neutralizing mAbs were checked by WB for the differentiation of the linear-neutralizing antigen site (site 1) and conformational antigen sites (sites 2–5) bound by these mAbs. The WB results showed that only mAb B66 (1/28) reacted with the bait antigen O/Mya/98 strain (Figure 4C) and recognized the linear C terminal in the VP1 of FMDV (Figures 4D,E). The remaining mAbs (27/28) were all negative by WB but obviously reacted with the FMDV antigen in IFA and ELISA (Figures 4A,B, Table S2). This result indicated that the immunodominant antigen site in cattle was dominated by non-linear epitopes rather than the linear epitope in antigen site 1 that was defined by conventional mouse-derived mAbs. For the surveying of the dominance of the antigen sites bound by 13 intra-type bnAbs, a panel of 20 serum samples from experimentally infected and vaccinated cattle was used in blocking ELISA for the testing of competitiveness with the 13 intra-type bnAbs. The results showed that the 13 intra-type bnAbs competed with all the tested serum samples, with a mean titer of >1:128, suggesting that these intra-type bnAbs all recognized dominant epitopes located in the antigenic structure of FMDV serotype O (Figure 5). By analyzing the titer frequencies of each bnAb corresponding to the tested serum samples, relatively high values were observed for bnAbs C4 (652 ± 71) and B77 (655 ± 243). Given the minor standard error of mean (SEM), the antigen site recognized by bnAb C4 might be the most immunodominant as this site elicited a relatively high and even antibody titer among different individuals.

### Ultralong HCDR3 Appeared in FMDV Serotype O-Neutralizing and Non-neutralizing IgG Antibodies, but Longer HCDR3 Biased for the Neutralizing IgG Antibodies

**Figure 6:**
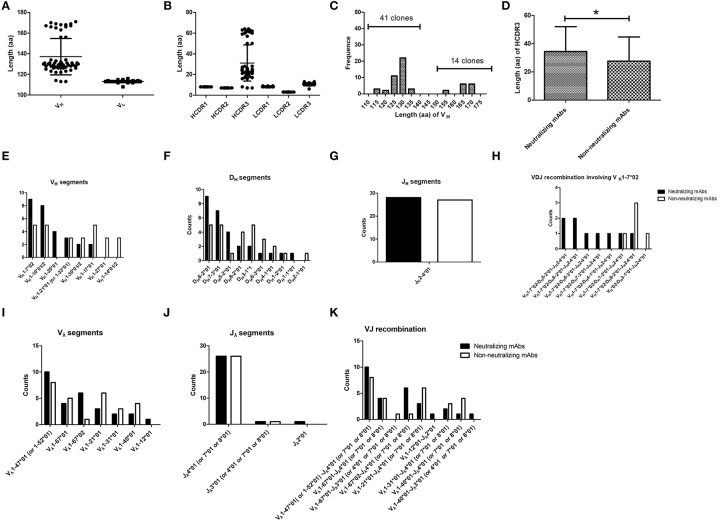
The CDRs and germline gene usage of FMDV-specific mAbs. Of the 55 FMDV-specific mAbs, the length of the VH and VL sequences **(A)** and the six CDRs **(B)**, as well as the frequency of the different length of HCDR3 **(C)** were analyzed. The HCDR3 length between the neutralizing and non-neutralizing FMDV-specific antibody clones **(D)** was examined by the Mann-Whitney test (one-tailed, *P* = 0.0419; ^*^P < 0.05). Data were presented as the mean ± standard error. To determine the individual gene segments employed by V-D-J rearrangements, the VH and VL sequences were separately aligned with bovine reference germline gene segments using the online IMGT/V-QUEST program. The counts of different gene segments or recombinations were displayed for V_H_ **(E)**, D_H_ **(F)**, J_H_ **(G)**, V_**λ**_ **(I)**, and J_**λ**_ **(J)** gene segments, V_H_1-7^*^02 containing V_H_-D_H_-J_H_ recombinations **(H)** and V_**λ**_-J_**λ**_ **(K)** recombinations of the neutralizing and non-neutralizing FMDV-specific antibody clones.

The sequences of variable domains of the 55 FMDV-specific mAbs were compared and analyzed. Every mAb was unique and harbored somatic mutations. The VH was indeed variable in length, ranging from 113 to 170 aa. VL was relatively similar in length (Figure 6A). Six complementary-determining regions (CDRs) from the VH and VL sequences of the 55 FMDV-specific mAbs were identified using the IMGT numbering scheme. We found that the diversity of VH was mainly caused by amino acid replacement and length alteration in HCDR3, which were 7–64 aa in length, whereas the other CDRs in the VH and VL of different clones were similar in length, although amino acid replacements appeared in these regions (Figures 6A,B). Notably, the proportion of ultralong HCDRs with more than 45 aa was 26% (Figure 6C; 14/55) and significant difference in HCDR3 length was observed between the neutralizing and non-neutralizing mAbs at statistical level (Figure 6D; *P* < 0.05). As revealed by mAb F145 with the ultralong HCDR3, which showed most potent neutralizing activity against all four representative FMDV strains, suggesting a relationship between HCDR3 length and the neutralizing breadth and potency against FMDV. These results suggest that cattle antibodies with longer HCDR3 are more inclined to display FMDV-neutralizing capacity and also suggest an involvement of ultralong HCDR3 in recognizing diverse virus antigens.

### Restricted Germline Gene Segments of V_H_ 1-7^*^02 and V_λ_ 1-47^*^01 (or 1-52^*^01) Pairing Were Observed in All Cattle IgG Antibodies With Ultralong HCDR3s

**Figure 7:**
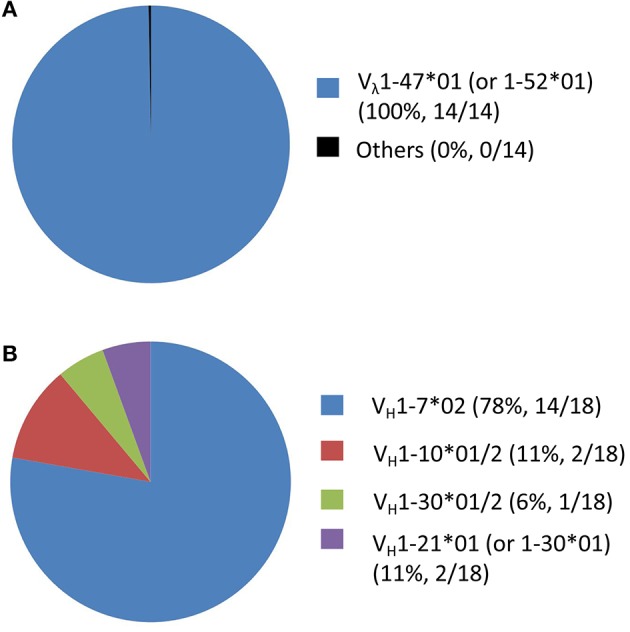
The pairings involved in the V_λ_1-47^*^01 (or 1-52^*^01) containing IgG antibodies as well as the V_H_1-7^*^02 containing IgG antibodies. The V_**λ**_ gene segments **(A)** in the IgG antibodies pairing with V_H_1-7^*^02 (n=18) were all (100%) V_λ_1-47^*^01 (or 1-52^*^01). Inversely, the proportion of V_H_ gene segments **(B)** in the IgG antibodies pairing with V_λ_ 1-47^*^01 (or 1-52^*^01) (*n* = 18) was 78% for V_H_1-7^*^02, 11% for V_H_1-10^*^01/2, 6% for V_H_1-30^*^01/2 and 6% for V_H_1-21^*^01 (V_H_1-33^*^01).

The usage of cattle IgG germline gene segments, the junction sequences and the somatic hypermutation counts of the FMDV-specific mAbs are listed in Table 3. All these antibody sequences exhibited somatic hypermutations and higher non-synonymous than synonymous mutations, as the observed amino acid rate was higher than the nucleotide mutation rate in both VH and VL sequences. At the genomic level, cattle Ig germline V(D)J gene rearrangements produced 37 unique V_H_-D_H_-J_H_ recombinations in the γ heavy chain and nine unique V_**λ**_-J_λ_ recombinations in the λ light chain (Figure 6K), which paired into 55 FMDV-specific mAbs. All the FMDV-specific mAbs were highly diverse and covered eight V_H_ gene segments (i.e., V_H_ 1-7^*^02; V_H_ 1-10^*^01/2; V_H_ 1-20^*^01; V_H_ 1-21^*^01 (or V_H_ 1-33^*^01); V_H_ 1-30^*^01/2; V_H_ 1-17^*^01; V_H_ 1-27^*^01 and 1-14^*^02), 10 D_H_ gene segments (i.e., D_H_ 6-2^*^01; D_H_ 7-3^*^01; D_H_ 5-2^*^01; D_H_ 8-2^*^01; D_H_ 3-1^*^01; D_H_ 6-3^*^01; D_H_ 4-1^*^01; D_H_ 1-2^*^01; D_H_ 1-1^*^01 and D_H_ 2-1^*^01), 1 sole J_H_ gene segment (J_H_ 2-4^*^01) for heavy chain (Figures 6E–G) and 7 V_λ_ and 3 J_**λ**_ gene segments for light chain (Figures 6I,J). Notably, all the cattle IgG antibodies that contained ultralong HCDR3 (total 14) were restricted to using the V_H_ 1-7^*^02 gene segment. Moreover, γ heavy chain containing the germline gene segment of V_H_ 1-7^*^02 was exclusively paired with the λ light chain containing germline gene segments of V_λ_1-47^*^01 or 1-52^*^01, and this was observed in all the cattle IgG antibodies with ultralong HCDR3 (Figure 7A). In contrast, the paring with V_λ_ 1-47^*^01 or 1-52^*^01 containing light chain was not solely restricted to γ heavy chain with the V_H_1-7^*^02 gene segment, as other V_H_ gene segments, such as V_H_1-10^*^01/2, V_H_1-30^*^01/2, and V_H_1-21^*^01 (V_H_1-33^*^01), are also involved in the pairing, accounting for 11, 6, and 6%, respectively (Figure 7B).

**Table 3:** Characterization of germline gene segments of the 55 FMDV-specific cattle mAbs.

**mAb**	**V_H_**	**D_H_**	**J_H_**	**Heavy-chain junction sequence**	**Mut^a^**	**V_λ_**	**J_λ_**	**Lambda-chain junction sequence**	**Mut^a^**
**A19**	1-21*01 or 1-33*01	7-3*01	2-4*01	CVK FFS GGW TYS CYG IDF GSV DAW	15 (11)	1-21*01 or 1-67*01	4*01 or 7*01 or 8*01	CAA GDD SSS SVD VF	8 (5)
**B77**	1-21*01 or 1-33*01	7-3*01	2-4*01	CAK SRY TGD GSI GLY GVD AW	22 (13)	1-31*01	4*01 or 7*01 or 8*01	CLSWQSGNTALF	17 (8)
**C9**	1-21*01 or 1-33*01	7-3*01	2-4*01	CVK CAN DYG SYF CYN YDY GYD FYV DAW	18 (13)	1-67*02	4*01 or 7*01 or 8*01	CVT YEL GSG AVF	32 (17)
**B57**	1-20*01	7-3*01	2-4*01	CVR LGA TKF GDY GCY GYG LYV DTW	26 (13)	1-67*01	4*01 or 7*01 or 8*01	CAA DDI VSR TT FF	19 (12)
**B66**	1-20*01	1-1*01	2-4*01	CGK LSG TGV CEG GCA CGH DPH VD AW	34 (15)	1-67*02	4*01 or 7*01 or 8*01	CVT YDS TIS AA VF	26 (16)
**B82**	1-20*01	3-1*01	2-4*01	CAK SSS GLW DGG CCG GST SCY VD SW	22 (11)	1-67*02	4*01 or 7*01 or 8*01	CVTYESSISTALF	18 (11)
**E18**	1-20*01 or 1-21*01 or 1-33*01	6-2*01	2-4*01	CAK DLC GGR ING CYN DGY YYS LRV DAW	31 (16)	1-21*01, or 1-67*01	4*01 or 7*01 or 8*01	CAA SDY SSN TV VF	9 (5)
**C5**	1-10*01 or 1-10*02	6-2*01	2-4*01	CAK SSR GVG WGD GLH YNE NDA GAW	20 (11)	1-12*01	2*01	CAS ADP NF	8 (6)
**F28**	1-10*01 or 1-10*02	6-2*01	2-4*01	CAK AAR GYA FTC GGI SSH SDH YVD AW	24 (12)	1-31*01	4*01 or 7*01 or 8*01	CAS WQS DST TVF	18 (7)
**F41**	1-10*01 or 1-10*02	6-2*01	2-4*01	CAK SYG YSG DGC NDD TYW YSD SYV DVW	15 (13)	1-67*01	4*01 or 7*01 or 8*01	CAS ADD SSN IA VF	18 (12)
**F53**	1-10*01 or 1-10*02	6-2*01	2-4*01	CGK WYN TGN YGC SKG YGN FDN YIE TW	26 (14)	1-67*01	4*01 or 7*01 or 8*01	CSS DDY ITT SA VF	25 (15)
**B73**	1-10*01 or 1-10*02	7-3*01	2-4*01	CAK LNS GGY GID GNG CYA FGS NYN LYV DAW	12 (8)	1-67*02	4*01 or 7*01 or 8*01	CTA YDH SIS TA VS	16 (9)
**B55**	1-10*01 or 1-10*02	5-2*01	2-4*01	CAK SNY WYY SCA ATN MYL DSW	10 (8)	1-40*01	3*01 or 4*01 or 7*01 or 8*01	CAC YDI NDN FD LF	25 (14)
**E34**	1-10*01 or 1-10*02	3-1*01	2-4*01	CAK CPS RWG CDY CDN YDFW	24 (12)	1-47*01 or 1-52*01	4*01 or 7*01 or 8*01	CAS ADD SSS NA VF	13 (6)
**F169**	1-10*01	8-2*01	2-4*01	YYC VKC IDS WCN YDD SGD IDAW	89 (39)	1-67*01	4*01 or 7*01 or 8*01	CAT GDY SLR TA VF	22 (14)
**B83**	1-7*02	5-2*01	2-4*01	CTN VHQ KTT TER SCP DLG YKY ECG NNC CWY SSC RGC IQG TYT STY NFY VH AW	8 (4)	1-47*01 or 1-52*01	4*01 or 7*01 or 8*01	CAS TED SSS NV VF	2 (2)
**F150**	1-7*02	5-2*01	2-4*01	CTT VHQ ETH TRK TCP DGYS NRA LPG CVK TCS YRD CCR FDR AGC RAS DYS VAY TYD FHV EAW	14 (8)	1-47*01 or 1-52*01	4*01 or 7*01 or 8*01	CAS AED SSS NA VF	3 (1)
**E54**	1-7*02	6-2*01	2-4*01	CGA VYQ TTE TKT TCP EGY SNT GDC DDD CCC WGS DCS RYA RWK RYR GGW FSS DYI VTE VYE FHV DAW	20 (10)	1-47*01 or 1-52*01	4*01 or 7*01 or 8*01	CAS AED SSS NV VF	4 (4)
**F166**	1-7*02	6-2*01	2-4*01	CTT VHQ ETK KSC SND YHY RYD CGE YVD CNE GNC CCS YAS GYC SWC NFR RVS PSY TYE HHV EAW	24 (13)	1-47*01 or 1-52*01	4*01 or 7*01 or 8*01	CAS AED ISN KF VF	8 (4)
**B74**	1-7*02	4-1*01	2-4*01	CAT VHQ ETK KSC SDG YYY RNE CGA YGD CTV GNC CCS YAS DYC NWC DFR RVT PTY TYE HHV EAW	13 (6)	1-47*01 or 1-52*01	4*01 or 7*01 or 8*01	CAS AED SSN NA VF	5 (4)
**F103**	1-7*02	1-2*01	2-4*01	CAI VHQ ETV RKT SGS DAY TCP DGC VLS PAC SRE RRC LCG TWP RDY CVD HIQ SST YNF YVE AW	20 (8)	1-47*01 or 1-52*01	4*01 or 7*01 or 8*01	CAS ADG SSS NA IF	6 (5)
**E46**	1-7*02	6-3*01	2-4*01	CST IEQ ETE RTT EKG CPE SCE GAF DCG HVP SYG RCA CCS WGT GTL YCC GTP RET YTY KWY VD AW	17 (11)	1-47*01 or 1-52*01	4*01 or 7*01 or 8*01	CAS AED SSR NA VF	8 (5)
**F145**	1-7*02	7-3*01	2-4*01	CTT VYH ETS RTC PDG YIY DPG CGG SWV CSR LFP TDR CIV GRT TTY EWY VD AW	7 (3)	1-47*01 or 1-52*01	4*01 or 7*01 or 8*01	CAS AED SSS NA VF	3 (2)
**E50**	1-7*02	8-2*01	2-4*01	CVT VHQ KSR DEK SCP DGY IDG AGC KYG WPC SDQ DCC VCS SCV YGY SGM NCV PAR YSE SYE WNV EAW	20 (9)	1-47*01 or 1-52*01	4*01 or 7*01 or 8*01	CAS AED SSS NA VF	5 (3)
**F128**	1-17*01	6-2*01	2-4*01	CAR CYS TCG CGL SCT SED SYY VN AW	15 (10)	1-67*02	4*01 or 7*01 or 8*01	CVT YDS TSS TIF	13 (10)
C4	1-17*01 or 1-39*03	6-2*01	2-4*01	CAK WSS RGG YDC GVH SSD YSY LD AW	30 (14)	1-67*02	4*01 or 7*01 or 8*01	CAA YDI STN AVF	18 (12)
A35	1-30*01 or 1-30*02	7-3*01	2-4*01	CAR SCG SYR DAW YDC ASD GYR YHN YVD AW	9 (6)	1-21*01	4*01 or 7*01 or 8*01	CAT ADY SSS TV VF	7 (7)
F136	1-30*01 or 1-30*02 or 1-30*03	5-2*01	2-4*01	ARE LYN GGS TWD AIN GYN EER YYF DAW	103 (44)	1-40*01	4*01 or 7*01 or 8*01	CAS PDS SSS GYF AVF	13 (6)
62	1-14*01	3-1*01	2-4*01	CAL GNY WAW	18 (9)	1-40*01	4*01 or 7*01 or 8*01	CAA YDI NGN AVF	15 (10)
A7	1-17*01	4-1*01	2-4*01	CVK SYW DYN DYG CCS GGN GVG FD AW	13 (6)	1-21*01	4*01 or 7*01 or 8*01	CAT ADY SRS TA VF	8 (4)
B51	1-10*01 or 1-10*02	4-1*01	2-4*01	CVK EHD NYG DFS GGC LHA AYV DTW	18 (9)	1-67*02	4*01 or 7*01 or 8*01	CVA YDS SSD SA IF	14 (8)
B54	1-30*01 or 1-30*02	6-3*01	2-4*01	CMR VGS CFG CGD RCG YGY PYT YVD VW	12 (5)	1-31*01	4*01 or 7*01 or 8*01	CAS YQI GNT AVF	2 (1)
B81	1-27*01	3-1*01	2-4*01	CVK LSR ESA WLF FHV DAW	28 (15)	1-67*01	3*01 or 4*01 or 7*01 or 8*01	CAG GDE NII VP LF	15 (9)
E28	1-21*01 or 1-33*01	7-3*01	2-4*01	CLR LAC YDH EGY RCF GYD LNW GVD AW	24 (14)	1-21*01	4*01 or 7*01 or 8*01	FCV TYD STI TPS AVF	63 (33)
E32	1-17*01 or 1-20*01	3-1*01	2-4*01	CAR DSG IYG TSG WGC IGG FDD NYI DAW	28 (14)	1-67*01	4*01 or 7*01 or 8*01	CAT SDY STR SS AF	19 (10)
E53	1-10*01 or 1-10*02	7-3*01	2-4*01	CAK ATD GGY FRS TYG CQG FTV NTY VD IW	17 (10)	1-67*01	4*01 or 7*01 or 8*01	CGS ADY SSE IA VF	26 (17)
60	1-14*02	6-3*01	2-4*01	CTG GGI GF IW	31 (19)	1-31*01	4*01 or 7*01 or 8*01	CAS YER NNT GVF	18 (10)
B59	1-21*01 or 1-33*01	6-2*01	2-4*01	CAK YFR HDY DVG CSY IME AVD AW	19 (11)	1-40*01	4*01 or 7*01 or 8*01	CAV WDD NIR NA VF	17 (9)
B64	1-10*01 or 1-10*02	7-3*01	2-4*01	CAK FFG DYG YDY YGC GYG AGD HYV DAW	14 (11)	1-47*01 or 1-52*01	4*01 or 7*01 or 8*01	CAS AEG SSS NA GF	4 (1)
E9	1-17*01	5-2*01	2-4*01	CTK CHY PGG CCG YWN DDH VD AW	16 (7)	1-21*01 or 1-67*01	4*01 or 7*01 or 8*01	CTT ADY SSS TV VF	11 (7)
E10	1-27*01	7-3*01	2-4*01	CTK VYN GGC GRR GYD AAA YVD AW	20 (11)	1-21*01	4*01 or 7*01 or 8*01	CAT ADY SSG TA VF	10 (5)
E11	1-17*01	6-2*01	2-4*01	CVK ESG SGY WDD ACW GFG VGD DYV DTW	20 (11)	1-40*01	4*01 or 7*01 or 8*01	CAV YDT SSK AA VF	18 (9)
E12	1-14*01	6-3*01	2-4*01	CTR CYE DYY YDC IDW GHR YD LW	16 (10)	1-31*01	4*01 or 7*01 or 8*01	CTS YES DYT AVF	9 (8)
E16	1-7*02	1-2*01	2-4*01	CSI VYQ KRE RKC PDG YRP GTF CGS GIN ARD CRY DGC YAS EHW QCC DTY TPG TSA YNF HID AW	23 (11)	1-47*01 or 1-52*01	4*01 or 7*01 or 8*01	CAS AEG SSS NA VF	11 (5)
E24	1-7*02	8-2*01	2-4*01	CAT VHQ ETG ERS CPV GSD CGG GCL HGC PCN ALT REW CRG DGV ERG GPC VCY PYF YTY EHH IE AW	19 (9)	1-47*01 or 1-52*01	4*01 or 7*01 or 8*01	CAS AED SSS NA VF	5 (3)
E40	1-7*02	8-2*01	2-4*01	CTT VHQ KTN TAK TCR DGH VDV SSC YGS SGC PRS GCC ACR RWG GTA CSI CSS RIV TYT YEF HVD VW	12 (5)	1-47*01 or 1-52*01	4*01 or 7*01 or 8*01	CAS PED SST NA LF	4 (2)
E43	1-7*02	3-1*01	2-4*01	CTV VHQ ETR QEE GCP DGY LYD SRCG PGG GCS GRL CTR TPS ARA NDF CCT GRR IRT STY QHH ID AW	21 (12)	1-47*01 or 1-52*01	4*01 or 7*01 or 8*01	CAS AED GNS NA VF	12 (6)
F4	1-21*01 or 1-33*01	3-1*01	2-4*01	CAK CGG FYG STC NGY GSS YD FW	21 (12)	1-47*01 or 1-52*01	4*01 or 7*01 or 8*01	CAS AED SSS NA VF	1 (0)
F36	1-17*01	7-3*01	2-4*01	CAK TTY SGD SRT FYG CYG SGS AYE TYV DTW	12 (4)	1-21*01	4*01 or 7*01 or 8*01	CGT ADD SSS IA VF	10 (9)
F39	1-10*01 or 1-10*02	6-2*01	2-4*01	CGK TSR FYG LVC NVD FYD DSE YVD AW	9 (6)	1-40*01	4*01 or 7*01 or 8*01	CAS PVG LGS GYP IF	31 (15)
F45	1-30*01 or 1-30*02	8-2*01	2-4*01	CVR CFS GYS PNS VCS TAD YVD AW	29 (16)	1-47*01 or 1-52*01	4*01 or 7*01 or 8*01	CAS AED SST NA VF	20 (11)
F56	1-30*01 or 1-30*02	6-2*01	2-4*01	CTT GFD LDC DWG YED STW	11 (7)	1-21*01	4*01 or 7*01 or 8*01	CAS ADD NTS TA VF	14 (12)
F64	1-27*01	2-1*01	2-4*01	CAT YVG DFW	21 (13)	1-67*01	4*01 or 7*01 or 8*01	CAA DDN SNS TA VF	9 (7)
F104	1-10*01 or 1-10*02	6-2*01	2-4*01	CTK SYG GND VYD CYD SE YW	16 (10)	1-67*01	4*01 or 7*01 or 8*01	CAT NDY SSD TT IF	8 (4)
F115	1-7*02	8-2*01	2-4*01	CTT THQ ISR KEQ RCP DGC RVN GWW GDS GCD DDT YCR YNY WGN CIR CTY VYT YEF HVD AW	18 (9)	1-47*01 or 1-52*01	4*01 or 7*01 or 8*01	CAS AED TSS NA VF	10 (5)

a *Mut, mutation number; the number of nucleotide mutations in the heavy and light chain variable domains and the number of amino acid replacements (is shown in parentheses). Bold font indicates the FMDV-neutralizing mAbs*.

*V_H_, variable gene segment of the heavy chain variable domain; D_H_, diversity gene segment of the heavy chain variable domain; J_H_, joining gene segment of the heavy chain variable domain; V_λ_, variable gene segment of the lambda light-chain variable domain; J_λ_, joining gene segment of the lambda light-chain variable domain*.

We further analyzed the usage of D_H_ gene segment in the 14 HCDR3 (150-192nt) sequences from nine FMDV-neutralizing mAbs and five non-neutralizing mAbs. As shown in Figure 6G, eight D_H_ gene segments, namely, D_H_ 8-2^*^01, D_H_ 3-1^*^01, D_H_ 6-2^*^01, D_H_ 7-3^*^01, D_H_ 5-2^*^01, D_H_6-3^*^01, D_H_ 4-1^*^01, and D_H_ 1-2^*^01, were used in the ultralong HCDR3s. Given that these eight D_H_ gene segments were only 13–23 nt in length that matched with their ultralong HCDR3s, the non-templated nucleotide-addition (also known as N-nucleotides) thus mainly contributed to the appearance of the ultralong HCDR3.

## Discussion

In this study, fully cattle-derived FMDV-neutralizing mAbs were successfully produced and characterized by a method combining the FACS isolation of antigen-specific single B cell, RT-PCR amplification of IgG antibody genes from single B cell and expression of antibody in CHO cells. This method is simple, convenient and feasible, and avoided complex hybridoma fusion and screening approaches. It thus represents an acceptable alternative for the development of diverse mAbs from a natural host.

The single B cell antibody approach has several unique advantages: (1) it harbors potential in isolating reactive functional mAbs against conformational determinants that can only be produced in natural hosts; (2) it facilitates the rapid screening of the correct clone efficiently and thus represents an approach that reduces time- and labor-intensive efforts and (3) it allows the screening of bnAbs that represent important tools in developing universal antiviral pharmaceuticals. To date, the generation of a mAb from plasmablasts was mainly described in humans (21), mice (22), rats (23), and rabbits (24). Recently, a pioneering study reported the possibility of obtaining mAbs from cattle plasmablasts by using this method and a model antigen derived from HIV (9). Our study used FMDV as the antigen and confirmed that the method of mAb production in cattle is feasible and efficient.

Generation of a diverse mAb repertoire is of great importance for different host species to respond specifically to countless antigenic challenges. Several mechanisms, including V-D-J recombination, somatic gene conversion and templated or untemplated somatic hypermutation, are fundamental to mAb repertoire diversification in mammals. Compared with human and mouse chromosomes, cattle chromosome 21 has a single IGH genetic locus family, which is inherently less variable and consists of 12 functional V_H_ gene segments, 23 D_H_ gene segments, and four functional J_H_ gene segments (http://www.imgt.org/IMGTveterinary/). In the obtained 55 mAbs sequences, all the heavy chain sequences corresponding to V-D-J germline genes displayed variable degrees of mutation at the nucleotide and amino acid level, and different lengths of N-nucleotides insertion at the V-D junction and/or D-J junction positions were observed in these mAbs. These results indicated that the sorted FMDV-specific plasmablasts showed somatic hypermutation, and all were mature B cells.

Sixteen functional D_H_ genes had been reported from 23 D_H_ gene segments in cattle and were divided into nine subgroups based on the length and identity of the coding sequence (25). It had been speculated that all members of D_H_ 2 and D_H_ 5 subgroups were pseudogenes because of divergent recombination signal sequences (25). However, we isolated one and five correct coding sequences in the D_H_ 2 and D_H_ 5 subgroups, respectively (Figure 6F), which confirmed that D_H_ 2 and D_H_ 5 subgroups were functional. Cattle Ig have an ultralong HCDR3, which is thought to compensate for the limited diversity of V-D-J recombination (7). The proportion of mAbs with ultralong HCDR3s was slightly higher than a previous observation (~9%) in bovine B cell hybridomas derived from mitogen-activated peripheral blood B cells (26). This discrepancy could result from the different methods used for isolation of the single B cell in cattle.

In this study, we observed the common phenomenon of ultralong HCDR3 appearing in the V_H_ sequence that was associated with the V_H_1-7^*^02 germline gene segment. This observation further confirmed previous descriptions (27, 28). A previous report indicated that restricted V_H_ and V_λ_ pairing appeared in ultralong HCDR3 containing IgM antibodies derived from fetal and adult cattle B cells without antigen stimulation (29). Our study extends these results by finding that restricted V_H_ and V_λ_ pairing also exists in cattle IgG antibodies after the antigen-stimulated μ heavy chain (IgM) isotype switching. In previous studies, long HCDR3s greatly contributed to antigen binding activity in several potent human bnAbs against HIV and influenza virus (30, 31). The characteristics of ultralong HCDR3 showed by anti-FMDV bnAbs in this study were consistent with those of bovine-derived bnAbs against HIV (9).

However, in this study, no clear evidence showed that the neutralization breadth and potency of FMDV-neutralizing mAbs are related to ultralong HCDR3 in cattle, although four intra-type bnAbs (E46, E54, F145, and F150) indeed displayed an ultralong HCDR3. Thus, selecting more FMDV strains to check the neutralization breadth and potency of these clones displaying ultralong HCDR3 could further determine the role of ultralong HCDR3 in the immune response of cattle against FMDV infection.

The screening of diverse neutralization mAbs will provide abundant tools for defining the antigenic structures of FMDV type O. Antigenic profiling as defined by mouse mAbs supported the existence of five neutralizing antigen sites on FMDV serotype O. Cattle-derived FMDV mAbs were previously developed by a mouse × cattle (lymph-derived B cell) hetero-hybridoma technique, and eight neutralizing mAbs were found to recognize identical antigen sites 2, 3 and 4 but not antigen sites 1 and 5 (16). Our study showed that most of the obtained cattle mAbs (27/28) bound to the conformational epitopes outside of antigen site 1. This may represent a significant feature of humoral immunity of cattle against FMDV. We need to further map the exact antigen sites recognized by these mAbs. However, the present data can only confirm that the immunodominant epitopes of FMDV serotype O responded by natural hosts are conformational epitopes rather than linear epitopes. Thus, comparing the data from different laboratories might provide an approach for improving understanding of the immunodominant antigenic structure of FMDV in natural hosts.

Due to the error-prone transcription of RNA-dependent RNA polymerase, and the high RNA recombination rate *in vivo*, the continuing circulation of FMDV in the field resulted in the emergence of many antigenic and genetic variants (32, 33). Comparative analysis of the epitopes revealed by these strain-specific mAbs can explain the antigenic variation of FMDV serotype O among ME-SA, SEA and Cathay topotypes. Currently, vaccination is the cheapest method for disease control in FMDV-endemic countries. However, new antigenic variants emerge periodically and likely contributed to vaccine failure. These intra-type bnAbs provide abundant tools for the identification of conserved epitopes and thus potentially provide references for the design of a universal vaccine against FMDV.

Apart from the resolution of FMDV antigenic structure, cattle-derived mAbs are endowed with a wide array of potential applications. These applications can be the serotyping of virus antigens with improved specificity, measurement of neutralizing antibody titers by competitive or blocking ELISA based on bnAbs, and quantification of virus antigens in inactivated vaccines as a quality control measure. Due to the natural features of these mAbs derived from cattle, it will afford diagnostic methods good performance with improved specificity and sensitivity.

In summary, we produced diverse FMDV-specific mAbs from cattle by single B cell antibody technique and mapped the corresponding cattle germline V, D, and J gene segments. The diversity of FMDV-specific mAbs was mainly arisen from somatic mutations, N-nucleotides insertion and germline V(D)J genes recombination. The restricted usage of germline gene segment in the ultralong HCDR3 containing antibodies were also observed in these FMDV-specific mAbs. The obtained FMDV-neutralizing mAbs from cattle predominately recognized conformational epitopes on the capsid surface of FMDV serotype O. This study provided a novel method for preparing FMDV-specific mAbs repertoire from the natural host, and these mAbs will be fundamental tools for exploring protective antigenic structure and variation.

## Materials and Methods

### Infection of Cattle

Three 1-year-old healthy *Qinchuan* cattle (*Bos taurus*), a Chinese breed of beef cattle, were raised in an Animal Bio-safety Level-3 laboratory for the sorting of antigenic specific antibody-secreting B cells after FMDV infection. Three cattle, designated as #2334, #1217, and #0005, were sequentially infected with the three topotypes of FMDV serotype O. The cattle were first challenged subcutaneously at two sites on the tongue with 10,000 BID_50_ (50% bovine infective dose) of cattle-adapted FMDV O/Mya/98 (GenBank JN998086). Before boost immunization, partially purified FMDV antigen without inactivation was formulated in Montanide ISA 201 oil-adjuvant (Seppic, Shanghai, China) in a 50:50 volume ratio. Then, each cattle received a boost vaccination with FMDV O/HN/CHA/93 (shares high homology with O/GD/CHA/86 GenBank AJ131468) and FMDV O/Tibet/99 (GenBank AJ539138) on days 35 and 132, respectively. Serum samples were collected at days 0, 7, 14, 21, 28, and 35 after primary infection and once per week or month after boost vaccination. Heparinized peripheral blood was taken from the jugular veins of the cattle for the isolation of PBMCs at various time points following immunization with a long span from initial day 102 to last day 225. All the animal experiments in the present study were approved by the Review Board of Lanzhou Veterinary Research Institute, Chinese Academy of Agricultural Sciences (Permit No. LVRIAEC2018-006) and conducted in accordance with the Animal Ethics Procedures and Guidelines of the People's Republic of China on animal use.

### Identification of FMDV-Specific Plasmablasts in Infected Cattle

PBMCs were isolated from the heparinized blood samples of the three cattle with HISTOPAQUE 1.083 (Sigma-Aldrich, USA) according to the manufacturer's instructions, and then used in the identification of FMDV-specific plasmablasts. Highly purified FMDV O/Mya/98 (FMDV) inactivated 146S antigen was biotinylated with EZ-Link™ NHS-LC-Biotin reagent (Thermo Fisher Scientific, USA) according to the manufacturer's instructions, and the resulting biotin-FMDV 146S in combination with anti-biotin APC were used for the staining of FMDV-specific plasmablasts (34). For staining, freshly isolated PBMCs were first stained with biotin-FMDV 146S, anti-bovine CD21-PE (Bio-Rad, USA) and anti-bovine IgM-FITC (Bio-Rad, USA, labeled with FITC in-house) for 30 min at 4°C in PBS buffer containing 2 mM EDTA and 0.5% BSA. Then, a second step antibody, mouse anti-biotin APC (Miltenyi Biotec, Germany), was added and incubated for 20 min at 4°C. The parallel staining of PBMCs that lacked biotin-FMDV 146S was used as fluorescence minus one (FMO) control. These stained samples were immediately analyzed by flow cytometry and one million PBMCs were acquired for counting the proportion of FMDV-specific plasmablasts.

### Single-Cell Sorting of FMDV-Specific Plasmablasts From Cattle PBMCs Using Flow Cytometry

Cattle PBMCs stained as above description were sorted by flow cytometry (BD FACS Aria II, USA) using a 100 μm nozzle. Cells were sorted for FMDV 146S-APC^+^/IgM-FITC^−^/CD21-PE^+/−^ events. Before the sorting of a single cell, 10 μl/well of single cell lysis buffer containing 1 μl of DNase (ThermoFisher Scientific, USA) was added into full skirt 96-well plates (Brand, Germany), then the targeted single cells were sorted at one cell per well by a BD FACSAria Fusion™ cell sorter. Immediately, the 10 μl/well of SuperScript™ VILO™ Master Mix (Thermo Fisher Scientific, USA) containing random primers were added for the synthesis of cDNA. After 60 min incubation at 42°C, the reaction was terminated at 85°C for 5 min. The cDNA templates were stored in −20°C for subsequent PCR amplification.

### Single-Cell PCR Amplification and Cloning of Variable Region Genes of IgG

The amplification of antibody variable region genes was performed by nested PCR using primers for cattle IgG gamma and lambda chains (Ig γ and Ig λ; Table 1). Given the λ chain accounting for 95% of cattle light chains, only the λ light chain was amplified by PCR. Two nested PCR amplifications were run per well for the λ light and γ heavy chains. The first PCR reaction was set up in 25 μl volume with 3 μl cDNA template and outer primer pairs (Table 1) using HotStar Taq Master Mix (Qiagen, Germany). The first round PCR begin with an initial hot-start at 95°C for 5 min, followed by 35 cycles of 95°C for 30 s (denaturation), 55–60°C for 50 s (annealing temperature listed in Table 1) and 72°C for 1 min (elongation), and ended with a final 5 min elongation at 72°C. The second nested PCR was performed as described above, and reaction was set up in a total volume of 50 μl with 5 μl of the first round PCR product as the template and inner primer pairs (Table 1). The final PCR product was Sanger sequenced. The paired heavy and light chain variable domain genes were synthesized by GenScript Inc. (www.genscript.com) with codon optimisation for expression in CHO cells, then cloned into cattle antibody expression vectors (CH-pcDNA3.4 and CL-pcDNA3.4 prepared in house; see Supplemental File 2), respectively, which contained cattle IgG2 heavy and light chain constant regions (CH and CL) with a tandem Myc and His tags at their respective C-terminus. These cloned antibody-expressing plasmids were proliferated in *E. coli* and then extracted and purified using an EndoFree Maxi plasmid kit (TIANGEN, China).

### Analysis of the Germline V(D)J Gene Segments Recombination in Cattle IgG Heavy and Light Chains

The usage of cattle Ig germline V_H_, D_H_ and J_H_ and V_λ_ and J_λ_ gene segments and the length of CDRs in heavy and light chains were analyzed by IMGT/V-QUEST program (Version 3.5.13) in the internet server (http://www.imgt.org/IMGT_vquest/input) (35, 36). This freely available program describes the mutations in VH and VL, hot spot positions in the closest germline V gene and insertions and deletions in the submitted sequences by alignment with the germline Ig gene and allele sequences of the IMGT reference directory (37), as well as the definition of CDRs based on IMGT unique numbering scheme (38).

### Full Cattle-Derived Antibody Production and Purification

The full cattle-derived antibodies were expressed in ExpiCHO-S™ cells (Invitrogen, USA) following the instruction of the manufacturer. Briefly, the 2–3 × 10^5^ cells/ml of CHO cells were suspended in 25 ml of serum free media in 125 mL flasks that were fixed in an orbital shaker platform at a speed of 125 rpm with a 25 mm shaking diameter. The cells were cultured at 37°C with ≥80% relative humidity and 8% CO_2_. After 3 days of culture, CHO cells at concentration of 6 × 10^6^ cells/ml were collected and resuspended in fresh media for transfection. The antibody expressing plasmids for light chain and heavy chain at a ratio of 3:2 were co-transfected into ExpiCHO-S™ cells with an ExpiFectamine™ CHO transfection kit (Invitrogen, USA), then the enhancer and feed component were added at 18 h post-transfection according to the standard protocol. Antibody-containing supernatants were harvested 10 days after transfection. The expressed mAbs were firstly purified by a HisTrap™ excel column, and the obtained elution was concentrated using a 100 kDa ultrafiltration tube and then further purified by size exclusion chromatography using Superdex 200 increase 10/300 column in an AKTA plus protein purification system (GE Life Sciences, Piscataway, NJ, USA). The purity and size of each full-length cattle IgG2 mAb were assessed by reducing and non-reducing SDS-PAGE. The concentrations of the final obtained IgG2 mAbs was determined by measuring their corresponding absorption values at a wavelength of 280 nm (A280).

### Virus Neutralizing Test

The cattle-derived antibodies were titrated for viral neutralizing activity against four representative strains from the three topotypes of FMDV serotype O by using a micro-neutralization assay as previously described (39). The four strains of FMDV were O/Mya/98 (SEA topotype), O/HN/CHA/93 (Cathay topotype), O/Tibet/99 (PanAsia lineage in ME-SA topotype), and O/XJ/CHA/2017 (India 2001 lineage in ME-SA topotype, GenBank No. MF461724). Briefly, antibody samples were 2-fold serially diluted in 96-well cell culture plates in a total volume of 50 μl, and then 100 TCID_50_ of FMDV in 50 μl of culture media was added to each well. After incubation for 1 h at 37°C, ~5 × 10^4^ BHK-21 cells in 100 μl media were added to each well as indicators of residual infectivity. Normal cell wells, and 10, 100, and 1,000 TCID_50_ virus control wells were used in each plate. The plates were incubated at 37°C under 5% CO_2_ conditions for 72 h before fixing and staining. The endpoint titers were calculated as the reciprocal of the last serum dilution to neutralize 100 TCID_50_ FMDV in 50% of the wells. Final antibody titer was expressed as IC_50_ calculated according to previously published method (9).

### Indirect Immunofluorescence Assay

BHK-21 cells in 24-well plates were infected with FMDV and then fixed in precooled methanol-acetone solution at a ratio of 1:1 (v/v) for 15 min at 4°C before appearance of cell lesions. After rinsing in PBS buffer, 200 μl/well cattle-derived antibodies at a concentration of 5 μg/ml were added and incubated for 1 h at 37°C. After washing with PBS buffer, FITC-rabbit anti-cattle IgG (Thermo-Fisher, USA) at a 1:1,000 dilution were added and incubated in the dark for 1 h at 37°C. Plates were washed three times with PBS and observed under an EVOS® FL Imaging System (Life Technology, USA).

### Enzyme-Linked Immunosorbent Assay

Liquid-phase blocking ELISA (LPB-ELISA) was used in the evaluation of antibody titers against FMDV after vaccination of cattle by a commercial LPB-ELISA kit from Lanzhou Veterinary Research Institute (LVRI; Lanzhou, Gansu, China) and performed according to the manufacturer's instructions (40).

Indirect ELISA was used in the assessment of the reactivity of cattle-derived antibodies with the FMDV 146S antigen. In indirect ELISA experiments, 200 ng/well of viral antigen was coated in 96-well plates overnight at room temperature. The plates were then washed three times with PBST (PBS buffer plus 0.05% Tween) and blocked with 1% gelatin in PBS at 37°C for 2 h. After three washes, the cattle-derived antibodies at a concentration of 1–10 μg/ml were added and incubated at 37°C for 1 h. The plates were washed three times with PBST, and then HRP-conjugated anti-His tag antibody (Genscript, China) at a dilution of 1:5,000 was added to the wells. The plates were then incubated at 37°C for 30 min and washed three times with PBST. Color was developed by adding 50 μl of TMB substrate (Pierce, Life Technology) for 10 min at room temperature. The process was stopped by adding equal volumes of 1 M H_2_SO_4_. Optical density at 405 nm (OD_450_) was measured on a microplate reader (BioRad).

Whether the expressed mAb can bind to a native and dominant epitope of virus particles by blocking with sera derived from FMDV infected or vaccinated cattle were determined by blocking ELISA. Briefly, the tested cattle mAbs were coated in a 96-well plate overnight, and the serum samples (PBS as control) were 2-fold serially diluted and incubated with a suitable concentration of FMDV 146S antigen for 30 min at room temperature and then transferred to an ELISA plate that was coated with mAbs for 1 h incubation at room temperature. After five washes with PBST, one HRP conjugated non-neutralizing mAb (HRP-B81) binding to FMDV capsid protein was added and incubated for 1 h. HRP-B81 antibody was used as an indicative antibody to show the capture of the virus capsid protein. After 5 washes in PBST, the color was developed and the reaction was stopped by the same procedure as previously described in indirect ELISA. The value corresponding to each tested mAb was expressed as the serum dilution that yielded 50% of the OD_450_ value of the PBS control. This value can in some extent reflect the dominance of the antigen site recognized by the tested mAb.

### Western-Blot

FMDV 146S antigen or GST fusion protein were subjected to 12% sodium dodecyl sulfate-polyacrylamide gel electrophoresis (SDS-PAGE). The protein bands in the gel were electro-transferred to a methanol-activated PVDF membrane followed by blocking with 5% non-fat milk in PBS overnight at 4°C. The membrane was probed with cattle-derived mAbs diluted to 2 μg/ml in PBS at 37°C for 1 h, followed by incubation with HRP-conjugated anti-His tag antibody that was diluted 1:4,000 at 37°C for 30 min. After thorough washing with PBST, the membrane was incubated with enhanced chemiluminescence reagent solution (Thermo Fisher Scientific) for 1 min and then exposed to X-ray film.

## Data Availability Statement

The sequences of all the cattle-derived monoclonal antibodies have been deposited in GenBank (Accession Numbers MN612644-MN612753).

## Ethics Statement

All cattle experiments were performed in a biosafety level 3 laboratory at Lanzhou Veterinary Research Institute (LVRI), Chinese Academy of Agricultural Sciences (CAAS). The study was approved by the Animal Ethics Committee of CAAS. The cattle were acclimated for at least 1 week before experimentation and humanely bred during the experiment. All cattle used in this study were euthanized at the end of the experiment.

## Author Contributions

KL and ZLu contributed to the overall concept and experimental design. KL and SW performed the cell sorting and gene amplification. SW performed the mAbs expression, purifications, and virus neutralizing test. YCa, PL, and YF performed the ELISA and IFA. HB, PS, and YCh prepared the viruses. XB and DL performed the vaccination and sampling. XL, FA, and FW prepared the inactivated antigen. XM and JZ participated the discussion and data analysis. KL drafted the manuscript. YCa reviewed the manuscript. ZLu and ZLi supervised the whole experiment. All authors read and approved the final manuscript.

### Conflict of Interest

XL, FA, and FW were employed by company China Agricultural Vet Biology and Technology Co. Ltd. The remaining authors declare that the research was conducted in the absence of any commercial or financial relationships that could be construed as a potential conflict of interest.

## References

[1] Mahapatra M Yuvaraj S Madhanmohan M Subramaniam S Pattnaik B Paton DJ. Antigenic and genetic comparison of foot-and-mouth disease virus serotype O Indian vaccine strain, O/IND/R2/75 against currently circulating viruses. Vaccine. (2015) 33:693–700. 10.1016/j.vaccine.2014.11.058 25500306 PMC4315132

[2] Mahapatra M Upadhyaya S Aviso S Babu A Hutchings G Parida S. Selection of vaccine strains for serotype O foot-and-mouth disease viruses (2007–2012) circulating in Southeast Asia, East Asia and Far East. Vaccine. (2017) 35:7147–53. 10.1016/j.vaccine.2017.10.099 29157957 PMC5720463

[3] Mccullough KC Crowther JR Butcher RN Carpenter WC Brocchi E Capucci L. Immune protection against foot-and-mouth disease virus studied using virus-neutralizing and non-neutralizing concentrations of monoclonal antibodies. Immunology. (1986) 58:421–8. 3015780 PMC1453459

[4] Mccullough KC De Simone F Brocchi E Capucci L Crowther JR Kihm U. Protective immune response against foot-and-mouth disease. J Virol. (1992) 66:1835–40. 1312607 10.1128/jvi.66.4.1835-1840.1992 PMC288969

[5] Stanfield RL Haakenson J Deiss TC Criscitiello MF Wilson IA Smider VV. The unusual genetics and biochemistry of bovine immunoglobulins. Adv Immunol. (2018) 137:135–64. 10.1016/bs.ai.2017.12.004 29455846 PMC5935254

[6] Saini SS Kaushik A. Extensive CDR3H length heterogeneity exists in bovine foetal VDJ rearrangements. Scand J Immunol. (2002) 55:140–8. 10.1046/j.1365-3083.2002.01028.x 11896930

[7] Haakenson JK Huang R Smider VV. Diversity in the cow ultralong CDR H3 antibody repertoire. Front Immunol. (2018) 9:1262. 10.3389/fimmu.2018.01262 29915599 PMC5994613

[8] Aitken R Hosseini A Macduff R. Structure and diversification of the bovine immunoglobulin repertoire. Vet Immunol Immunopathol. (1999) 72:21–9. 10.1016/S0165-2427(99)00113-0 10614489

[9] Sok D Le KM Vadnais M Saye-Francisco KL Jardine JG Torres JL. Rapid elicitation of broadly neutralizing antibodies to HIV by immunization in cows. Nature. (2017) 548:108–11. 10.1038/nature23301 28726771 PMC5812458

[10] Xie QC Mccahon D Crowther JR Belsham GJ Mccullough KC. Neutralization of foot-and-mouth disease virus can be mediated through any of at least three separate antigenic sites. J Gen Virol. (1987) 68:1637–47. 10.1099/0022-1317-68-6-1637 2438378

[11] Mccahon D Crowther JR Belsham GJ Kitson JD Duchesne M Have P. Evidence for at least four antigenic sites on type O foot-and-mouth disease virus involved in neutralization; identification by single and multiple site monoclonal antibody-resistant mutants. J Gen Virol. (1989) 70:639–45. 10.1099/0022-1317-70-3-639 2471793

[12] Kitson JD Mccahon D Belsham GJ. Sequence analysis of monoclonal antibody resistant mutants of type O foot and mouth disease virus: evidence for the involvement of the three surface exposed capsid proteins in four antigenic sites. Virology. (1990) 179:26–34. 10.1016/0042-6822(90)90269-W 1699353

[13] Crowther JR Farias S Carpenter WC Samuel AR. Identification of a fifth neutralizable site on type O foot-and-mouth disease virus following characterization of single and quintuple monoclonal antibody escape mutants. J Gen Virol. (1993) 74:1547–53. 10.1099/0022-1317-74-8-1547 8393912

[14] Aktas S Samuel AR. Identification of antigenic epitopes on the foot and mouth disease virus isolate O1/Manisa/Turkey/69 using monoclonal antibodies. Rev Sci Tech. (2000) 19:744–53. 10.20506/rst.19.3.1244 11107617

[15] Asfor AS Upadhyaya S Knowles NJ King DP Paton DJ Mahapatra M. Novel antibody binding determinants on the capsid surface of serotype O foot-and-mouth disease virus. J Gen Virol. (2014) 95:1104–16. 10.1099/vir.0.060939-0 24584474 PMC3983758

[16] Barnett PV Samuel AR Pullen L Ansell D Butcher RN Parkhouse RM. Monoclonal antibodies, against O1 serotype foot-and-mouth disease virus, from a natural bovine host, recognize similar antigenic features to those defined by the mouse. J Gen Virol. (1998) 79:1687–97. 10.1099/0022-1317-79-7-1687 9680132

[17] Kohler G Milstein C. Continuous cultures of fused cells secreting antibody of predefined specificity. Nature. (1975) 256:495–7. 10.1038/256495a0 1172191

[18] Steinitz M Klein G Koskimies S Makel O. EB virus-induced B lymphocyte cell lines producing specific antibody. Nature. (1977) 269:420–2. 10.1038/269420a0 198669

[19] Winter G Milstein C. Man-made antibodies. Nature. (1991) 349:293–9. 10.1038/349293a0 1987490

[20] Tiller T. Single B cell antibody technologies. Nat Biotechnol. (2011) 28:453–7. 10.1016/j.nbt.2011.03.014 21473940 PMC7102800

[21] Jin A Ozawa T Tajiri K Obata T Kondo S Kinoshita K. A rapid and efficient single-cell manipulation method for screening antigen-specific antibody-secreting cells from human peripheral blood. Nat Med. (2009) 15:1088–92. 10.1038/nm.1966 19684583

[22] Tiller T Busse CE Wardemann H. Cloning and expression of murine Ig genes from single B cells. J Immunol Methods. (2009) 350:183–93. 10.1016/j.jim.2009.08.009 19716372

[23] Ouisse LH Gautreau-Rolland L Devilder MC Osborn M Moyon M Visentin J. Antigen-specific single B cell sorting and expression-cloning from immunoglobulin humanized rats: a rapid and versatile method for the generation of high affinity and discriminative human monoclonal antibodies. BMC Biotechnol. (2017) 17:3. 10.1186/s12896-016-0322-5 28081707 PMC5234254

[24] Starkie DO Compson JE Rapecki S Lightwood DJ. Generation of recombinant monoclonal antibodies from immunised mice and rabbits via flow cytometry and sorting of antigen-specific IgG+ memory B cells. PLoS ONE. (2016) 11:e0152282. 10.1371/journal.pone.0152282 27022949 PMC4811437

[25] Ma L Qin T Chu D Cheng X Wang J Wang X. Internal duplications of DH, JH, and C region genes create an unusual IgH gene locus in cattle. J Immunol. (2016) 196:4358–66. 10.4049/jimmunol.1600158 27053761

[26] Saini SS Allore B Jacobs RM Kaushik A. Exceptionally long CDR3H region with multiple cysteine residues in functional bovine IgM antibodies. Eur J Immunol. (1999) 29:2420–6. 10458755 10.1002/(SICI)1521-4141(199908)29:08<2420::AID-IMMU2420>3.0.CO;2-A

[27] Berens SJ Wylie DE Lopez OJ. Use of a single VH family and long CDR3s in the variable region of cattle Ig heavy chains. Int Immunol. (1997) 9:189–99. 10.1093/intimm/9.1.189 9043960

[28] Lopez O Perez C Wylie D. A single VH family and long CDR3s are the targets for hypermutation in bovine immunoglobulin heavy chains. Immunol Rev. (1998) 162:55–66. 10.1111/j.1600-065X.1998.tb01429.x 9602352

[29] Saini SS Farrugia W Ramsland PA Kaushik AK. Bovine IgM antibodies with exceptionally long complementarity-determining region 3 of the heavy chain share unique structural properties conferring restricted VH + Vlambda pairings. Int Immunol. (2003) 15:845–53. 10.1093/intimm/dxg083 12807823

[30] Walker LM Phogat SK Chan-Hui PY Wagner D Phung P Goss JL. Broad and potent neutralizing antibodies from an African donor reveal a new HIV-1 vaccine target. Science. (2009) 326:285–9. 10.1126/science.1178746 19729618 PMC3335270

[31] Corti D Voss J Gamblin SJ Codoni G Macagno A Jarrossay D. A neutralizing antibody selected from plasma cells that binds to group 1 and group 2 influenza A hemagglutinins. Science. (2011) 333:850–6. 10.1126/science.1205669 21798894

[32] Domingo E Escarmis C Baranowski E Ruiz-Jarabo CM Carrillo E Nunez JI. Evolution of foot-and-mouth disease virus. Virus Res. (2003) 91:47–63. 10.1016/S0168-1702(02)00259-9 12527437

[33] Mahapatra M Parida S. Foot and mouth disease vaccine strain selection: current approaches and future perspectives. Expert Rev Vaccines. (2018) 17:577–91. 10.1080/14760584.2018.1492378 29950121

[34] Kenney M Waters RA Rieder E Pega J Perez-Filguera M Golde WT. Enhanced sensitivity in detection of antiviral antibody responses using biotinylation of foot-and-mouth disease virus (FMDV) capsids. J Immunol Methods. (2017) 450:1–9. 10.1016/j.jim.2017.07.001 28689695

[35] Brochet X Lefranc MP Giudicelli V. IMGT/V-QUEST: the highly customized and integrated system for IG and TR standardized V-J and V-D-J sequence analysis. Nucleic Acids Res. (2008) 36:W503–8. 10.1093/nar/gkn316 18503082 PMC2447746

[36] Giudicelli V Brochet X Lefranc MP. IMGT/V-QUEST: IMGT standardized analysis of the immunoglobulin (IG) and T cell receptor (TR) nucleotide sequences. Cold Spring Harb Protoc. (2011) 2011:695–715. 10.1101/pdb.prot5633 21632778

[37] Lefranc MP Lefranc G The Immunoglobulin Factsbook. London: Academic Press (2001).

[38] Lefranc MP Pommie C Ruiz M Giudicelli V Foulquier E Truong L. IMGT unique numbering for immunoglobulin and T cell receptor variable domains and Ig superfamily V-like domains. Dev Comp Immunol. (2003) 27:55–77. 10.1016/S0145-305X(02)00039-3 12477501

[39] Golde WT Pacheco JM Duque H Doel T Penfold B Ferman GS. Vaccination against foot-and-mouth disease virus confers complete clinical protection in 7 days and partial protection in 4 days: use in emergency outbreak response. Vaccine. (2005) 23:5775–82. 10.1016/j.vaccine.2005.07.043 16153756

[40] Cao Y Lu Z Li P Sun P Fu Y Bai X. Improved neutralising antibody response against foot-and-mouth-disease virus in mice inoculated with a multi-epitope peptide vaccine using polyinosinic and poly-cytidylic acid as an adjuvant. J Virol Methods. (2012) 185:124–8. 10.1016/j.jviromet.2012.03.036 22766183

