# Corrigendum: Development of Foot-and-Mouth Disease Virus-Neutralizing Monoclonal Antibodies Derived From Plasmablasts of Infected Cattle and Their Germline Gene Usage

**DOI:** 10.3389/fimmu.2020.00286

**Published:** 2020-02-21

**Authors:** Kun Li, Sheng Wang, Yimei Cao, Huifang Bao, Pinghua Li, Pu Sun, Xingwen Bai, Yuanfang Fu, Xueqing Ma, Jing Zhang, Dong Li, Yingli Chen, Xuerong Liu, Fanglan An, Faju Wu, Zengjun Lu, Zaixin Liu

**Affiliations:** ^1^State Key Laboratory of Veterinary Etiological Biology, Lanzhou Veterinary Research Institute, Chinese Academy of Agricultural Sciences, Lanzhou, China; ^2^China Agricultural Vet Biology and Technology Co. Ltd., Lanzhou, China

**Keywords:** cattle, foot-and-mouth disease virus, broadly neutralizing antibodies, antigenic character, single B cell antibody

In the original article, “**Kenney et al. (2017)**” was not cited. The citation has now been inserted in the **MATERIALS AND METHODS**, subsection **Identification of FMDV-Specific Plasmablasts in Infected Cattle**, paragraph one:

“PBMCs were isolated from the heparinized blood samples of the three cattle with HISTOPAQUE 1.083 (Sigma-Aldrich, USA) according to the manufacturer's instructions, and then used in the identification of FMDV-specific plasmablasts. Highly purified FMDV O/Mya/98 (FMDV) inactivated 146S antigen was biotinylated with EZ-Link^TM^ NHS-LC-Biotin reagent (Thermo Fisher Scientific, USA) according to the manufacturer's instructions, and the resulting biotin-FMDV 146S in combination with anti-biotin APC were used for the staining of FMDV-specific plasmablasts (34). For staining, freshly isolated PBMCs were first stained with biotin-FMDV 146S, anti-bovine CD21-PE (Bio-Rad, USA) and anti-bovine IgM-FITC (Bio-Rad, USA, labeled with FITC in-house) for 30 min at 4°C in PBS buffer containing 2 mM EDTA and 0.5% BSA. Then, a second step antibody, mouse anti-biotin APC (Miltenyi Biotec, Germany), was added and incubated for 20 min at 4°C. The parallel staining of PBMCs that lacked biotin-FMDV 146S was used as fluorescence minus one (FMO) control. These stained samples were immediately analyzed by flow cytometry and one million PBMCs were acquired for counting the proportion of FMDV-specific plasmablasts.”

The authors apologize for this error and state that this does not change the scientific conclusions of the article in any way. The original article has been updated.
