# Supplementary material for: Development of Foot-and-Mouth Disease Virus-Neutralizing Monoclonal Antibodies Derived From Plasmablasts of Infected Cattle and Their Germline Gene Usage
Source: Front Immunol. 2019 Dec 6;10:2870. doi: 10.3389/fimmu.2019.02870 (PMC6908506; doi:10.3389/fimmu.2019.02870)
Supplement: Supplementary file 1 [file Table_1.docx]

Supplemental

Table S1. Sera tested for neutralization against strains from 3 topotypes of O type FMDV at given time points after infection

| Cattle No. | ^a^ Strains | Day 0 | Day 1 | Day 4 | Day 7 | Day 14 | Day 21 | Day 28 | Day 35 | Day 42 | Day 49 | Day 56 | Day 63 | Day 132 | Day 169 | Day 223 |
| --- | --- | --- | --- | --- | --- | --- | --- | --- | --- | --- | --- | --- | --- | --- | --- | --- |
| #1217 | O/HN/CHA/93 | ﹤8 | ﹤8 | 128 | 256 | 128 | 64 | 45 | 22 | 90 | 512 | 512 | 360 | 360 | 256 | 360 |
|  | O/Tibet/99 | ﹤8 | ﹤8 | 128 | 360 | 256 | 256 | 64 | 256 | 256 | 360 | 360 | 90 | 16 | 512 | 512 |
|  | O/Mya/98 | ﹤8 | ﹤8 | 180 | 360 | 360 | 512 | 256 | 180 | 512 | ﹥512 | ﹥512 | ﹥512 | ﹥512 | ﹥512 | 512 |
| #2334 | O/HN/CHA/93 | ﹤8 | ﹤8 | 16 | 180 | 90 | 180 | 256 | 32 | 512 | 512 | 512 | 512 | ﹥512 | ﹥512 | 360 |
|  | O/Tibet/99 | ﹤8 | ﹤8 | 180 | 256 | 128 | 128 | 256 | 360 | ﹥512 | ﹥512 | ﹥512 | ﹥512 | ﹥512 | ﹥512 | 256 |
|  | O/Mya/98 | ﹤8 | ﹤8 | 90 | 256 | 360 | 360 | 512 | ﹥512 | ﹥512 | ﹥512 | ﹥512 | ﹥512 | ﹥512 | ﹥512 | ﹥512 |
| #0005 | O/HN/CHA/93 | ﹤8 | ﹤8 | 32 | 180 | 45 | 64 | 90 | 45 | 128 | ﹥512 | 360 | 512 | 512 | 360 | 64 |
|  | O/Tibet/99 | ﹤8 | ﹤8 | 90 | 512 | 256 | 256 | 256 | 360 | ﹥512 | ﹥512 | ﹥512 | ﹥512 | ﹥512 | ﹥512 | 256 |
|  | O/Mya/98 | ﹤8 | ﹤8 | 128 | 256 | ﹥512 | ﹥512 | 180 | 360 | 512 | ﹥512 | ﹥512 | ﹥512 | ﹥512 | ﹥512 | 512 |

^a^Neutralizing antibody titres are expressed as the max serum dilutions that neutralized 100 TCID_50_ of O/Mya/98 (SEA topotype), O/HN/CHA/09 (Cathay topotype) and O/Tibet/99 (PanAsia topotype) FMDV.
