# Supplementary material for: Development of Foot-and-Mouth Disease Virus-Neutralizing Monoclonal Antibodies Derived From Plasmablasts of Infected Cattle and Their Germline Gene Usage
Source: Front Immunol. 2019 Dec 6;10:2870. doi: 10.3389/fimmu.2019.02870 (PMC6908506; doi:10.3389/fimmu.2019.02870)
Supplement: Supplementary file 2 [file Table_2.docx]

Supplemental

Table S2. Information of the 55 FMDV-specific mAbs from 3 cattle plasmablasts

| ^a^ mAb | ^b^ IFA | ^c^ ELISA | ^d^ WB | ^e^ Isolation time | Cattle No |
| --- | --- | --- | --- | --- | --- |
| ***A19*** | **-** | **+** | **-** | 138 | #1217 |
| ***A35*** | ****** | **+** | **-** | 138 | #1217 |
| ***B55*** | **-** | **-** | **-** | 154 | #1217 |
| ***B57*** | ******* | **++++** | **-** | 154 | #1217 |
| ***E46*** | ****** | **+** | **-** | 223 | #0005 |
| ***B66*** | ******* | **++++** | **+** | 154 | #1217 |
| ***B73*** | ******* | **++++** | **-** | 154 | #2334 |
| ***B74*** | **-** | **+** | **-** | 154 | #2334 |
| ***B77*** | **-** | **+++** | **-** | 154 | #2334 |
| ***B82*** | ******* | **++++** | **-** | 154 | #2334 |
| ***B83*** | **-** | **++** | **-** | 154 | #2334 |
| ***C4*** | ******* | **++++** | **-** | 168 | #2334 |
| ***C5*** | **-** | **++** | **-** | 168 | #2334 |
| ***C9*** | ***** | **+** | **-** | 168 | #2334 |
| ***E18*** | ****** | **++** | **-** | 223 | #1217 |
| ***E34*** | **-** | **+** | **-** | 223 | #2334 |
| ***E50*** | **-** | **-** | **-** | 223 | #0005 |
| ***E54*** | ****** | **+** | **-** | 223 | #0005 |
| 62 | - | + | **-** | 102 | #1217 |
| A7 | *** | ++ | **-** | 138 | #1217 |
| B51 | ** | ++ | **-** | 154 | #1217 |
| B54 | ** | + | **-** | 154 | #1217 |
| B81 | *** | ++++ | **-** | 154 | #2334 |
| E28 | *** | ++++ | **-** | 223 | #2334 |
| E32 | *** | ++++ | + | 223 | #2334 |
| E53 | *** | ++++ | + | 223 | #0005 |
| 60 | - | ++ | **-** | 102 | #1217 |
| B59 | - | + | **-** | 154 | #1217 |
| B64 | * | + | **-** | 154 | #1217 |
| E9 | - | + | **-** | 223 | #1217 |
| E10 | - | ++++ | **-** | 223 | #1217 |
| E11 | - | + | **-** | 223 | #1217 |
| E12 | - | ++ | **-** | 223 | #1217 |
| E16 | - | ++ | **-** | 223 | #1217 |
| E24 | - | ++ | **-** | 223 | #2334 |
| E40 | - | ++++ | **-** | 223 | #2334 |
| E43 | - | ++++ | **-** | 223 | #0005 |
| F4 | - | + | **-** | 225 | #1217 |
| F36 | * | ++ | - | 225 | #1217 |
| F39 | * | ++ | - | 225 | #1217 |
| F45 | - | + | - | 225 | #1217 |
| F56 | - | + | - | 225 | #1217 |
| F64 | - | + | - | 225 | #1217 |
| F104 | *** | ++++ | + | 225 | #2334 |
| F115 | * | ++ | - | 225 | #2334 |
| ***F28*** | *** | ++++ | - | 225 | #1217 |
| ***F41*** | **-** | **-** | **-** | 225 | #1217 |
| ***F53*** | **-** | **-** | **-** | 225 | #1217 |
| ***F103*** | ******* | ++++ | **-** | 225 | #2334 |
| ***F128*** | ***** | **+** | **-** | 225 | #2334 |
| ***F136*** | **-** | **+** | **-** | 225 | #2334 |
| ***F145*** | **-** | **-** | **-** | 225 | #2334 |
| ***F150*** | **-** | **-** | **-** | 225 | #2334 |
| ***F166*** | **-** | **-** | **-** | 225 | #2334 |
| ***F169*** | ******* | **++++** | **-** | 225 | #2334 |

^a^ mAbs with virus neutralizing activity are indicated with bold italic letters .

^b^ IFA was performed in BHK cell infected with FMDV O/Mya/98, and the results were classified into high(***) , medium(**) and low(*) by fluorescence intensity to show the binding activity of mAbs to virus antigen. The “-” indicated the mAbs could not bound to virus antigen by IFA.

^c^ FMDV O/Mya/98 146S antigen were coated on ELISA plate, and the binding activity of mAbs were evaluated by indirect ELISA, and the two fold OD_450_ value of PBS control was used as the cut off value. MAbs reactivity in indirect ELISA were marked respectively as “++++”, “+++”, “++”, “+”, corresponding to the lowest concentration of each diluted mAb (ranged from 0-0.05 μg/ml, 0.05-0.1μg/ml, 0.1-1μg/ml and 1-5 μg/ml) that gave an OD_450_ value greater than cut off value.

^d^ Western-blot (WB) results, “+” and “-” means respectively positive and negative results in this assay.

^e^ Days after the first immunization.
