# Supplementary material for: Development of Foot-and-Mouth Disease Virus-Neutralizing Monoclonal Antibodies Derived From Plasmablasts of Infected Cattle and Their Germline Gene Usage
Source: Front Immunol. 2019 Dec 6;10:2870. doi: 10.3389/fimmu.2019.02870 (PMC6908506; doi:10.3389/fimmu.2019.02870)
Supplement: Supplementary file 3 [file Data_Sheet_1.docx]

**Supplemental Fig. S1.**


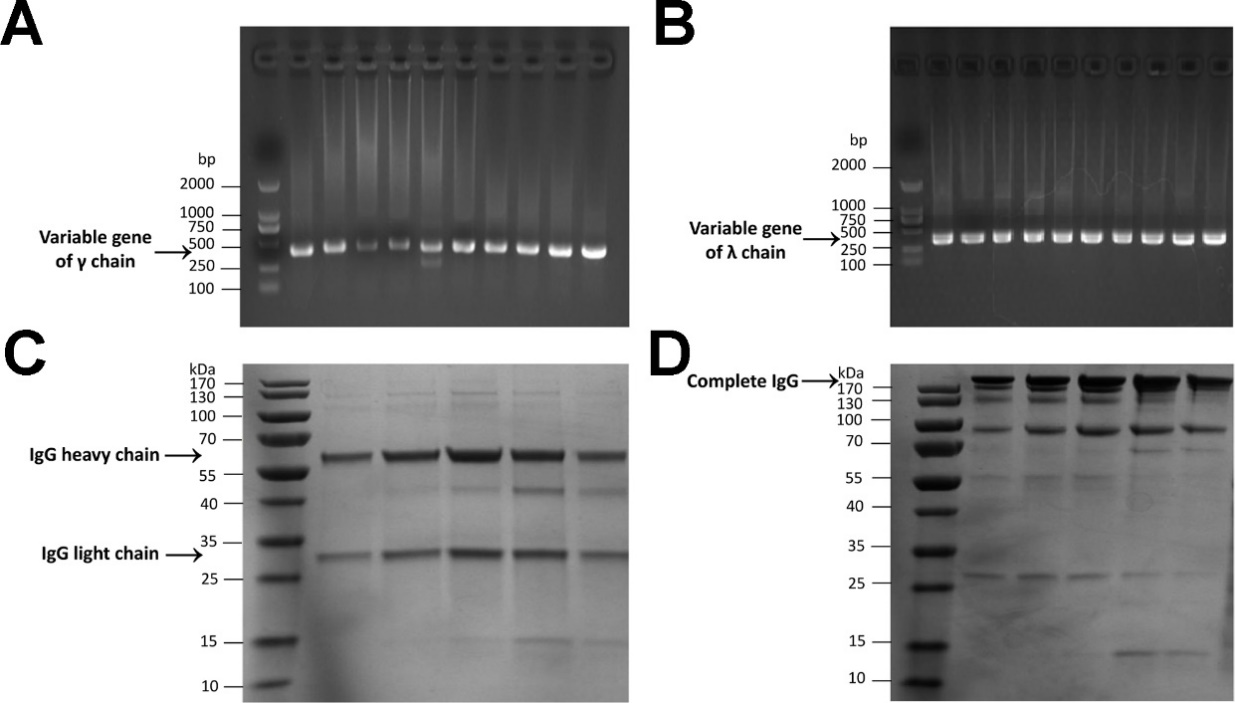


**Fig. S1. Nest PCR production of cattle VH and VL, and SDS-PAGE analysis of purified complete cattle IgG proteins under reduced or non-reduced condition.** Second round of PCR products for the variable genes of the heavy chain (A) and light chain (B) were confirmed by agarose gel electrophoresis. The purified IgG mAbs were boiled using 5×loading buffer with DTT (C) or without DTT (D) and then were respectively subjected to 12% SDS-PAGE.
